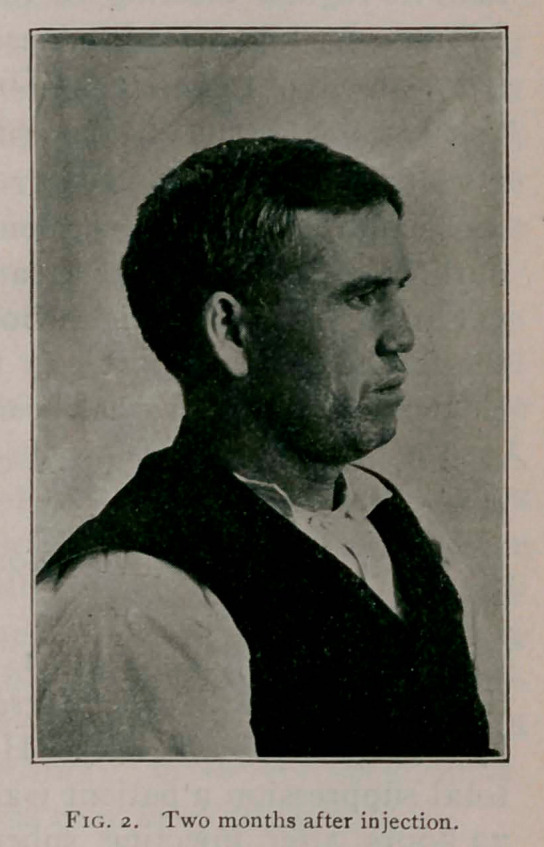# Subcutaneous Injection of Paraffin in the Correction of Nasal Deformity

**Published:** 1903-06

**Authors:** Edgar A. Forsyth

**Affiliations:** Buffalo, N. Y.; 322 Franklin Street


### CLINICAL REPORT.

### Subcutaneous Injection of Paraffin in the Correction of Nasal Deformity.

By EDGAR A. FORSYTH, M. D., Buffalo, N. Y.

IN 1900, Gersuny, of Vienna, began the use of paraffin for the correction of nasal deformities. He found that the paraffin became encapsuled and reported two cases, one of two years' standing with no visible changes in the contour of the nose nor any softening of the paraffin. He mentions also a case in which the patient passed through typhoid fever with a temperature as high as 104°F., and no disturbance of the paraffin resulted other than temporary softening.

The paraffin should have a melting point of 102°F. If it is above the body temperature, it will be carried away by the lymphatics; if too hard, it may cause necrosis. The lump paraffin is too hard and white vaseline is too soft. These two products I had melted together in proportion to secure the desired consistency, about the same as that of vaseline, with a melting point of 102°F. The paraffin is sterilised by heating it at its boiling point for a few minutes before using, which will kill all germs.

For the purpose of injection, I used an antitoxin syringe, filled with the melted paraffin, inverted, all the air forced out, and the needle screwed on. The instrument was placed in hot water until needed. Before making the injection the syringe should be tested to see that the needle is not obstructed; if it is, pass a wire through it. The paraffin is allowed to cool so that it will flow from the syringe in a semi-solid or worm-like condition.

The operation is not a painful one and therefore I did not use any local anesthetic. The only preparation of the patient was to cleanse the skin thoroughly before making the injection. The accompanying photographs, taken before and after the injection, show the result obtained.

P.D., aged 36, contracted syphilis when about 19 years old, which destroyed the cartilaginous septum and caused a sinking

FIG. 1. Before injection.

FIG. 2. Two months after injection.

in of the nose—saddle-back depression as it is commonly called. He said his nose had been deformed for about nine years. Operated on December 31, 1902, inserting needle from above and carrying the point to the lowest portion. As the injection was being made the needle was withdrawn and the paraffin was molded into the desired shape, while soft. While the injection was being made pressure was applied to the upper part of the nose to prevent the paraffin from running into the inner side of the nose and the canthus of the eyes. After a sufficient amount had been injected a compress was placed over the nose and kept on for twelve hours No reaction took place; little or no pain followed the injection.

322 FRANKLIN STREET.

---

THERAPY OF PURPURA HEMORRHAGICA.—Algin claims that hypodermoclysis of a normal salt solution (up to 2 pts.) proved very effective in arresting the bleeding and controlling the disease.—*International Medical Magazine.*

## Figures and Tables

**Fig. I. f1:**
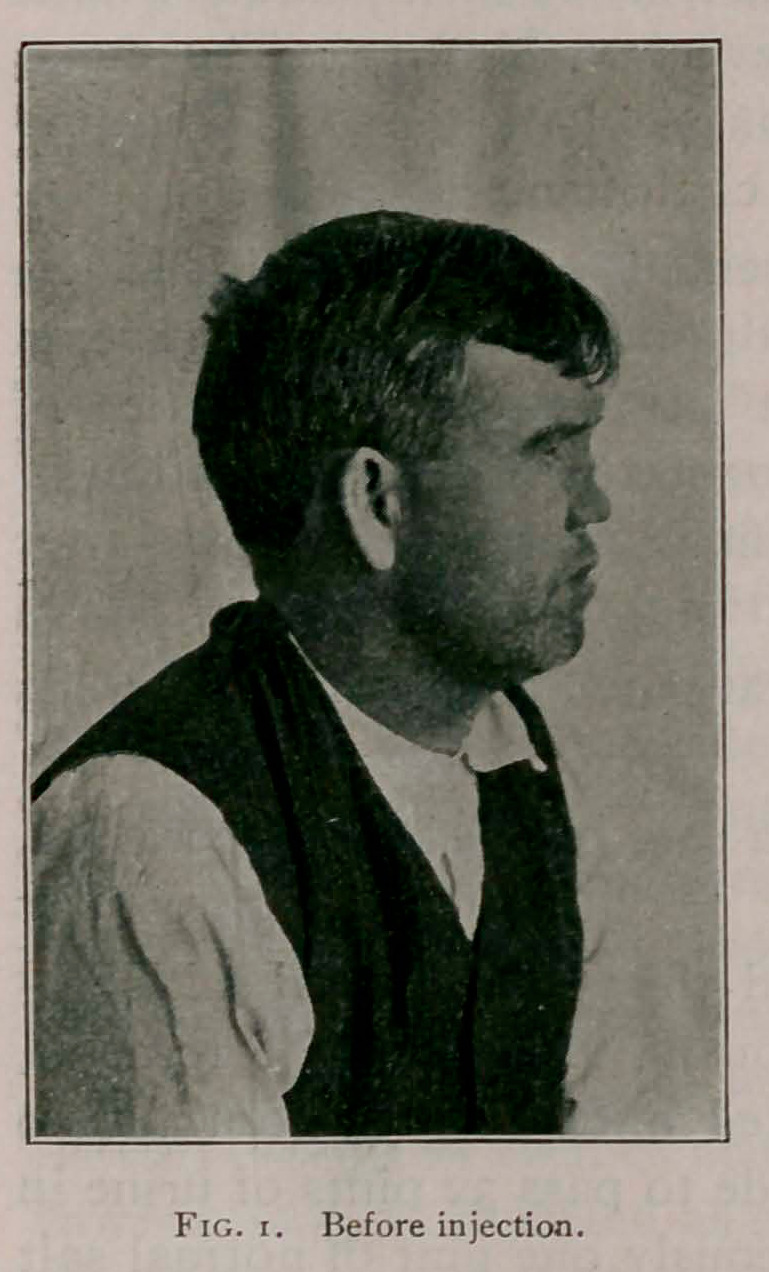


**Fig. 2. f2:**